# Estimation of respiratory rate from motion contaminated photoplethysmography signals incorporating accelerometry

**DOI:** 10.1049/htl.2018.5019

**Published:** 2019-02-21

**Authors:** Delaram Jarchi, Peter Charlton, Marco Pimentel, Alex Casson, Lionel Tarassenko, David A. Clifton

**Affiliations:** 1 Department of Engineering Science, Institute of Biomedical Engineering, University of Oxford, Oxford, UK; 2 School of Medicine, King's College London, London, UK; 3 School of Electrical and Electronic Engineering, University of Manchester, Manchester, UK

**Keywords:** accelerometers, medical signal processing, patient monitoring, health care, pneumodynamics, photoplethysmography, motion corrupted PPG signals, motion artefact, respiratory rate, motion contaminated photoplethysmography signals, motion reduction, simultaneous acceleration signals, accelerometer sensors, autoregressive based technique, PPG signals, reconstructed PPGs, home-based monitoring, motion states, rest states, accelerometry, Hilbert domain

## Abstract

Estimation of respiratory rate (RR) from photoplethysmography (PPG) signals has important applications in the healthcare sector, from assisting doctors onwards to monitoring patients in their own homes. The problem is still very challenging, particularly during the motion for large segments of data, where results from different methods often do not agree. The authors aim to propose a new technique which performs motion reduction from PPG signals with the help of simultaneous acceleration signals where the PPG and accelerometer sensors need to be embedded in the same sensor unit. This method also reconstructs motion corrupted PPG signals in the Hilbert domain. An auto-regressive (AR) based technique has been used to estimate the RR from reconstructed PPGs. The proposed method has provided promising results for the estimation of RRs and their variations from PPG signals corrupted with motion artefact. The proposed platform is able to contribute to continuous in-hospital and home-based monitoring of patients using PPG signals under various conditions such as rest and motion states.

## 1 Introduction

Respiratory rate (RR) is a key physiological measurement which can be combined with other vital signs to derive patients’ early warning scores [1]. Previous studies have shown that it is a highly informative indicator of adverse events such as clinical deterioration [2–6]. Common practice for monitoring patients’ RR is for nurses to count the number breaths in a minute. However, this is not always accurate or repeatable [7] and is done infrequently as part of 4 h manual observations. To provide more regular and accurate measurements, continuous monitoring of patients’ vital signs is possible through the use of non-obtrusive and light-weight wearable sensors. These signals are often corrupted by motion artefact, so robust signal processing and machine learning techniques are required to obtain reliable estimates. A wide range of existing algorithms have recently been introduced to estimate the RR from photoplethysmography (PPG) signals using finger probes or wrist-worn sensors. Other studies have been also proposed which obtain a PPG signal from standard video data [8–10]. However, the quality of the PPG data from wearable sensors (wrist-type PPG sensor or pulse oximeter with a finger/ear probe) is higher than video-based systems, so in this research wrist-type PPG sensor is used to derive RR.

Previous methods used in the estimation of RR from PPG or electrocardiogram (ECG) signals include joint time-frequency analysis [11, 12], the short time Fourier transform (STFT) [13], correntropy spectral estimates [14], AR models [15–17] and sparse signal reconstruction [18]. The method in [18] has shown to be able to estimate RR with a PPG signal sampled at 10 Hz, making it a potential application for low-cost wearable sensors. Recent studies propose using respiratory induced modulations of the PPG signal, which include the variation in amplitude, intensity and frequency [19, 20]. Estimates of RR from each of these are fused to get the final RR value [21–23]. A comprehensive review of existing algorithms to estimate RR from PPG or ECG signals has recently been introduced in [24].

Most of the mentioned methods are used to estimate RR for patients who are at rest. This is appropriate for sensors in intensive care units because there is much less movement, however for continuous in-hospital and home-based monitoring, it is necessary to estimate various vital signs from signals corrupted by motion artefact. In this work, signal processing techniques are demonstrated on a public dataset which has been used in many studies for robust estimation of heart rate (HR) under intense motion. This has particular applications for sports monitoring systems. Since the estimation of the RR during motion is very much limited in the literature, the principal aim of this Letter is to demonstrate a novel technique for obtaining the RR of subjects undertaking physical exercise. Since the proposed signal reconstruction technique aims to reconstruct pulse peaks, part of the method can be used for estimation of heart rate variability (HRV) from motion contaminated PPG signals in future studies. With more improvements in the PPG signal reconstruction stage, more reliable estimates for HRV can be obtained.

This Letter is organised as follows. Section 2 describes the methods used, it explains the proposed algorithms for reduction of motion artefact from PPG signals using simultaneous accelerometer signals, and then PPG signal reconstruction using the Hilbert transform (HT). The corresponding outputs are used to estimate the RR from an auto-regressive (AR) model [23]. In Section 3, the results for estimated RRs are provided. The toolbox proposed in [21] has been used to derive reference data for RR estimates using ECG signals, and these ECG-based estimates of RR are compared to the PPG-based results. Section 4 concludes the Letter by summarising the PPG and ECG-based results, as well as the main contributions of this Letter, the limitations of the study and potential future work.

## 2 Methods

### 2.1 Reduction of PPG motion artefact using simultaneous accelerometer signals

An analysis of the spectrum of simultaneous PPG and accelerometer signals recorded during physical exercise demonstrates that they have similar spectral components [25]. An adaptive filter has been used to reduce the motion interference from PPG signals, by removing these similar spectra. Motion-free PPG is recovered by using the accelerometer signals as inputs to a normalised least mean squares (NLMS) filter. The measured PPG signal $p\lpar t\rpar $ in the time domain can be modelled using the following equation:

(1) $$p\lpar t\rpar = \widetilde{\,p}\lpar t\rpar + m\lpar t\rpar + v\lpar t\rpar \eqno\lpar 1\rpar $$

where $\widetilde{\,p}\lpar t\rpar $ is a motion-free PPG signal, $m\lpar t\rpar $ is the motion artefact and $v\lpar t\rpar $ is the sensor noise. The NLMS filter assumes that the motion artefact in the PPG is linearly related to the accelerometer signal, $m\lpar t\rpar = {\bi h}^T\lpar t\rpar {\bi a}_{{\rm acc}}\lpar t\rpar $, ${\bi h}\lpar t\rpar $ is a vector of filter coefficients, with length *L*, and ${\bi a}_{{\rm acc}}\lpar t\rpar $ a vector of accelerometer signal at time *t* and the previous $L - 1$ time points. The error output of the filter, is therefore

(2) $$e\lpar t\rpar = p\lpar t\rpar - {\bi h}^T\lpar t\rpar {\bi a}_{{\rm acc}}\lpar t\rpar \eqno\lpar 2\rpar $$

The NLMS adaptive filter modifies its coefficients in order to minimise the mean squared error, using the linear update equation:

(3) $${\bi h}\lpar t + 1\rpar = {\bi h}\lpar t\rpar + \displaystyle{{\mu \lpar t\rpar } \over {{\Vert }{\bi a}_{{\rm acc}}\lpar t\rpar {\Vert }^2}}{\bi a}_{{\rm acc}}\lpar t\rpar e\lpar t\rpar \eqno\lpar 3\rpar $$

where $\mu \lpar t\rpar $ is the step size.

### 2.2 Reconstruction of PPG signal using the HT

Multiple NLMS filters can be used to remove motion from the PPG signal, each using a different accelerometer axis as its input. The filters’ outputs can be combined to produce an enhanced spectrum, e.g. by multiplying the output spectra of the NLMS filters [25, 26]. Based on this, the final spectrum can be used to track desired frequencies such as HR. However, the final spectrum is not useful for estimating RR, since low-frequency information relating to RR is lost due to the influence of motion artefacts which were likely removed by the NLMS filters. Here, the objective is to reconstruct the corresponding clean PPG signal using parts of the spectrum that are free of motion components. The reconstructed PPG signal can be used for two purposes:

- Estimation of peaks in the raw PPG signals to derive amplitude, intensity and frequency modulations which are used for the estimation of RR.
- Estimation of peaks in the raw PPG signal which are correlated with R peaks detected in the corresponding ECG signal. This has potentials for analysis of HRVs from PPG signals.

The focus of this Letter is on the estimation of RR. Estimation of HRV from PPG signals can be further discussed and improved in future studies.

To reconstruct the PPG signal, HT based technique has been used. This allows instantaneous amplitude, phase and frequency to be derived at all time points for input signals (each NLMS output signal), these can then be combined to reconstruct the final PPG signal. For each NLMS filter output, the analytic signal is formed using the signal as the real part and its HT as the imaginary part

(4) $$p\lpar t\rpar + {\rm j}{\tt H}\lpar p\lpar t\rpar \rpar = a\lpar t\rpar {\rm e}^{{\rm j}\theta \lpar t\rpar }\eqno\lpar 4\rpar $$

where ${\tt H}\lpar .\rpar $ denotes the HT operation, ${\rm j}$ is $\sqrt { - 1} $, $a\lpar t\rpar $ and $\theta \lpar t\rpar $ represent instantaneous amplitude and phase, respectively. The Hilbert spectrum is computed at each time point, considering the energy of the signal as the power of the instantaneous amplitude at the calculated instantaneous frequency. The input signal can be reconstructed using the real part of the complex analytic signal

(5) $$p\lpar t\rpar = \Re \lpar a\lpar t\rpar {\rm e}^{{\rm j}\theta \lpar t\rpar }\rpar \eqno\lpar 5\rpar $$

where $\Re \lpar .\rpar $ denotes the real part. The above equation can also be written

(6) $$p\lpar t\rpar = a\lpar t\rpar \cos \lpar \theta \lpar t\rpar \rpar \eqno\lpar 6\rpar $$

Therefore using analytic signal formed in (4) and the associated instantaneous amplitude and phase, it is possible to construct the original signal as the real part of the analytic signal. An important ability of the HT is to form the analytic signal and then construct the original signal. The instantaneous amplitudes and corresponding phases create the spectrum of the signal, therefore, they can be used to denoise or reconstruct the original signal (e.g. using thresholding, removing sparse values etc.). For reconstruction of motion free PPG signal, the instantaneous phases of the outputs of the *n* adaptive filters (*n* combination of PPG and accelerometer axis) given by the HT are fused to reconstruct a new signal

(7) $$\varphi \lpar t\rpar = \coprod\limits_{i = 1}^n {\cos \left({\displaystyle{{\theta _i\lpar t\rpar } \over n}} \right)\comma \; } \quad r\lpar t\rpar = \root n \of {\coprod\limits_{i = 1}^n {a_i\lpar t\rpar } } \cdot \hat \varphi \lpar t\rpar \eqno\lpar 7\rpar $$

where *n* is the number of NLMS filters, $a_i\lpar t\rpar $ and $\theta _i\lpar t\rpar $ represent instantaneous amplitude and phase, respectively, of the *i*th NLMS filter output. $r\lpar t\rpar $ is the reconstructed motion free PPG. Based on the above equation, signal $\hat \varphi \lpar t\rpar $ (the phase modulated signal) is used to construct $r\lpar t\rpar $ and detect peaks using peakdet function, (MATLAB, the MathWorks Inc.) with a threshold of 0.1 on $r\lpar t\rpar $, after amplification and normalisation of $\varphi \lpar t\rpar $ between −1 to 1. This normalisation of $\varphi \lpar t\rpar $ to obtain $\hat \varphi \lpar t\rpar $, is necessary to reconstruct the PPG signal since after dividing the phase by a factor of *n* as in (7), the corresponding cosine function will generate values between 0 and 1 rather than between −1 to 1. In case of having two signals with equal instantaneous phases and amplitudes, using (7), the reconstructed signal $r\lpar t\rpar $ will be proportional to original signals. Whilst it is not straightforward to demonstrate mathematically that (7) will reconstruct a noise-free signal. The idea behind this equation is to combine the spectrum given by all outputs of the adaptive filters to obtain a single amplified and enhanced spectrum where there is a shared spectral content among all filter outputs (by multiplication of instantaneous amplitudes and corresponding instantaneous phases) to reconstruct a motion reduced PPG. Detected peaks on reconstructed PPGs are expected to be highly correlated with R peaks detected on ECG signals. In addition, the detected peaks and onsets are tracked back to the raw PPG signal where they are used to derive respiratory induced modulations such as amplitude, intensity and frequency to estimate RR.

### 2.3 Estimation of RR from reconstructed PPG

To estimate RR in terms of the number of breaths per minute, a recent method based on the AR models has been applied to the reconstructed PPG signal. The proposed method in [23] fuses spectral estimates of three respiratory-induced modulations (intensity, amplitude and frequency) using multiple AR models. Intensity modulation is associated with variation in intrathoracic pressure leading to a change in the baseline of perfusion and is defined to reflect the variation in amplitude of the time-series of peak amplitudes of the PPG. Frequency modulation is related to the change in the instantaneous HR during the respiratory cycle, also known as respiratory-sinus arrhythmia and is calculated using the difference in the timing of consecutive pulse peaks in the PPG (which correspond to instantaneous HR). Amplitude modulation is related to changes in cardiac output related to the quantity of refill in the vessels at the periphery. It is defined to be the difference in peak amplitudes of consecutive peaks and troughs, effectively resulting in a time-series of the height of the PPG pulse. It has been suggested in [27], to obtain pulse width in order to derive respiration from PPG pulse. The corresponding respiratory modulation based on pulse width can be explored in future studies.

To derive amplitude modulation, a time series relating to amplitudes of pulse peaks (the difference between the height of consecutive peak and onset) needs to be constructed while only the peak amplitudes of the pulses are required to derive the intensity modulation. To derive the frequency modulation, a time series presenting the difference between the timing of consecutive pulse peaks need to be formed. The median spectrum of these models is used to estimate the RR by finding the maximum peak in the spectrum in [23]. Based on this, a median spectrum across all respiratory modulations is calculated considering various model orders. The AR spectra with less peakedness have a lower weight in the calculated median spectrum and, therefore, less contribution in the estimation of final respiratory frequency.

**Fig. 1:**
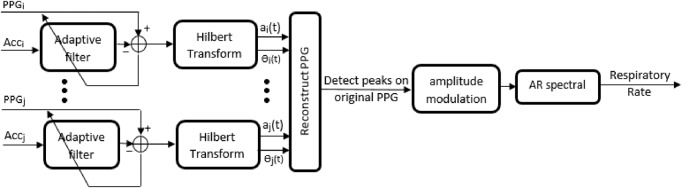
Block diagram of the proposed method for estimation of RR from PPGs and simultaneous accelerometers. In the last stage, GP Regression has been applied to smooth the resulted RRs

This method has produced highly accurate RR estimates for PPG signals obtained from subjects at rest. Here, we apply a similar version of the method to the reconstructed PPG signals. In this Letter, instead of fusing respiratory frequencies from three different modulations, only amplitude modulations have been used in the experiment. Therefore, the amplitude modulated signal representing pulse heights is generated and resampled to a 4 Hz signal and then it is subjected to the AR spectral analysis to estimate RRs. These are in strong agreement with the estimates which used intensity modulations mainly for ECG signals. The block diagram of the proposed method is summarised in Fig. 1.

In another recent Letter, a public RR toolbox has been proposed by Charlton *et al.* [21] to estimate RR using 314 algorithms for ECG and PPG signals. The algorithms are divided into three stages: first, the respiratory signals are extracted, then the main RR is estimated, followed by the fusing of RR estimates. The techniques for fusing RR estimates include smart fusion [22] (using baseline wander, amplitude modulation and frequency modulation as respiratory signals), spectral peak-conditioned averaging [28], pole magnitude criterion [16], pole ranking criterion [29] and temporal smoothing [27]. The toolbox has been extensively validated against reference data on various datasets. In this work, the toolbox has been used to create the reference data by combining RR estimates from ECG based methods whose outputs strongly agree. It has also been used to obtain PPG based estimates for comparison with the results of our proposed method. For pairwise comparison, the RR toolbox has been used considering only amplitude modulated signals. In the last stage temporal fusion has been performed, so that smoother results were obtained from the motion corrupted PPG/ECG signals.

### 2.4 Participants and data characteristics

The dataset used in this research is a publicly available dataset [30, 31] which has been recently used in a large number of studies to estimate average and instantaneous HR during motion [25]. The dataset provides raw signals including a single channel ECG recorded from the chest, two PPG signals using green light reflected from the wrist and accelerometer signals from three axes embedded in the same wristband sensor unit as the PPG sensors, The data was collected from 12 male subjects aged 18–35 who were walking/running on the treadmill for about 5 min in the following order of speeds: the speed of 1–2 km/h for 0.5 min, the speed of 6–8 km/h for 1 min, the speed of 12–15 km/h for 1 min, the speed of 6–8 km/h for 1 min, the speed of 12–15 km/h for 1 min, and the speed of 1–2 km/h for 0.5 min. The sampling frequency of all the signals is set to 125 Hz. Although the dataset has been extensively used for estimating HR, it has not been evaluated for estimation of RR. The dataset has been used in this Letter to estimate RR using various algorithms and to derive the ground truth data.

### 2.5 Reference data

The dataset selected in this Letter includes average HR estimates as a reference and raw signals of several simultaneous recordings from different sensors. The accelerometer sensor has been shown to be especially useful in modelling the contaminated motion artefact of PPG signals. However, one limitation in using this dataset to estimate RR is the lack of a reference data for RR estimates in order to evaluate the accuracy of the proposed technique. To obtain reference data, the public RR toolbox [21] has been used to provide a reference for RR estimates using ECG signals. The ECG signals are expected to produce more accurate RR estimates than PPGs [21]. That is the reason to select several ECG based methods in our Letter to derive the reference data from RR toolbox. Here, we have applied the RR toolbox using only amplitude modulated signals. In the following detailed explanations are provided to derive the reference data.

## 3 Results

### 3.1 Reduction of motion artefact and PPG reconstruction

**Fig. 2:**
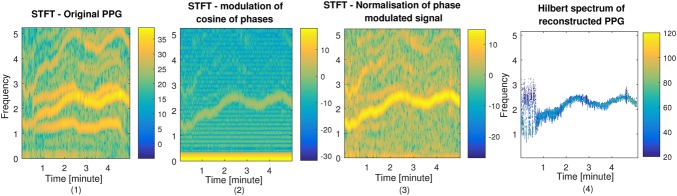
Motion reduction and PPG signal reconstruction stages (1–4) in spectral domain for subject 8 *1* STFT of the raw PPG signal for subject 8 *2* STFT of the extracted phase modulated signal *3* STFT of the amplified and normalised phase modulated signal *4* Hilbert spectrum of the reconstructed PPG signal

**Fig. 3:**
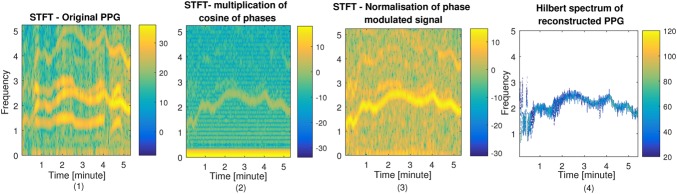
Motion reduction and PPG signal reconstruction stages (1–4) in spectral domain for subject 9 *1* STFT of the raw PPG signal for subject 9 *2* STFT of the extracted phase modulated signal *3* STFT of the amplified normalised phase modulated signal *4* Hilbert spectrum of the reconstructed PPG signal

To reduce motion artefact of the PPG signals, NLMS filters as explained in Section 2.1 have been applied to the two PPG signals, using each of the three accelerometer axis signals. Therefore, for each subject six NLMS filters are designed. For each NLMS filter, the $\mu $ and the filter order *L* have been set to 0.1 and 9, respectively. For subjects 8 and 9 from the dataset, the time-frequency spectrum is shown in Figs. 2 and 3, respectively. The time–frequency spectra of raw PPG signals affected by motion artefact is shown in Figs. 2*a* and 3*a*, using the STFT with window length 6 s with an overlap of 2 s. As can be seen from Figs. 2 and 3, the motion related spectral components in Figs. 2*a* and 3*a* are diminished in Figs. 2*b* and 3*b* where only the dominant HR frequency is visible. This is crucial for PPG signal reconstruction stage.

After applying the HT to the output of each adaptive filter (using Hilbert function, MATLAB, The MathWorks, Inc.), the STFT of $\varphi \lpar t\rpar $ in (7) is shown in Figs. 2*b*, 3*b*, 2*c* and 3*c*, before and after signal normalisation and amplification, respectively. Finally, the Hilbert spectrum of the reconstructed PPG signal $r\lpar t\rpar $ (see (7)) is shown in Figs. 2*d* and 3*d* for subjects 8 and 9, respectively. The Hilbert spectrum has been computed at each time point using instantaneous amplitude and frequency for motion reduced reconstructed PPG as a narrow band signal. The STFT has been computed for a window of 6 s long to present the time–frequency spectrum of motion corrupted PPG signals (as a broad band signal) and also their extracted phase modulated signals. The reconstructed PPG signal is expected to contain the main HR frequency. Based on the peaks detected in the reconstructed PPG signal, HRV can be measured. The reconstructed PPG signals can be used to estimate HRV using intervals between detected R peaks. Estimation of HRV from motion contaminated PPG signals [25, 32] has not been extensively studied yet. Therefore, the proposed signal reconstruction in Hilbert domain can be used to improve HRV in future studies. Here the reconstructed PPG signal is subjected to the AR-based model to estimate RR.

### 3.2 Estimation of RR

**Fig. 4:**
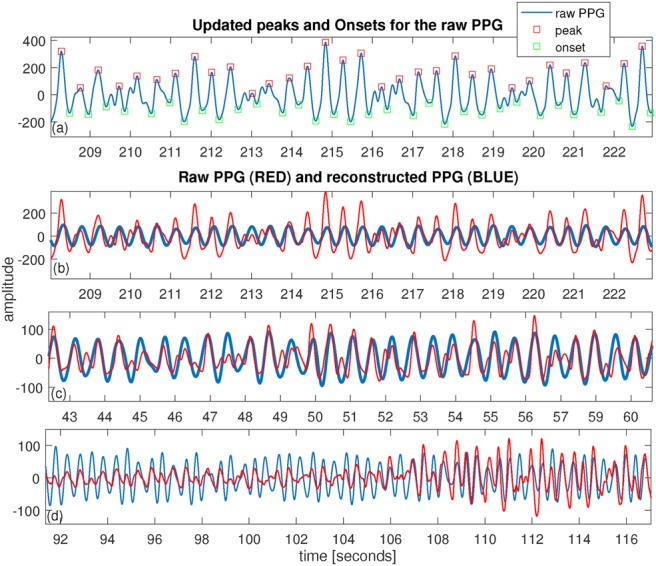
Examples of raw and reconstructed PPGs *a* Raw PPG signal and detected peaks and onsets *b* The same data segment as in (a) where both raw PPG and reconstructed PPG are shown *c*, *d* Two selected segments of the data during motion including raw PPG and reconstructed PPG

For estimation of RR, the recent method based on the AR model [23] has been applied to the reconstructed PPG signals. Since the raw PPG signals are very noisy in the time domain, detection of pulse peaks would be impossible in most cases. Therefore, it is more reliable to detect peaks on the reconstructed PPG than on the raw signal (see Fig. 4). Thus for estimation of RR, the peaks and onsets of the reconstructed PPG are located back into the raw PPG signals using a small window size. This will help to better extract the amplitude modulated signal used to estimate RR since after PPG reconstruction low-frequency information and signal amplitude could be affected (see Fig. 4). Using the described dataset, subject number 11 was removed in the analysis as the ECG signals show saturation in the recordings. For the 11 subjects remaining in the dataset, the RR has been estimated by applying the AR spectral-based method [23] to derived amplitude modulations from reconstructed PPGs. For the AR model, instead of selecting an optimal model order, AR models with varying model orders $p = 2\comma \; ...\comma \; 19$ are applied. Then, a median AR spectrum has been calculated that is a more enhanced spectrum and the maximal peak is used as a potential RR frequency. To do this, data segments of length 64 s with an overlap of 60 s are used to estimate RRs. To smooth the RR estimates, Gaussian process (GP) regression [33] with a Matern covariance function, has been used where the hyperparameters are set to 0.25 and 1 for the characteristic length scale and standard deviation, respectively. The constant value of the Matern covariance related to the smoothness of the GP has been set to three. In addition, the Gaussian likelihood function has been applied while the mean function has not been used. The minimise cost function that minimises the negative log marginal likelihood in the GP allowed 50 function evaluations. To estimate RR from the ECG signal, manual peak detection has been performed on the raw ECG signals, then the AR-based method has been used to estimate RR using the extracted amplitude modulated signal.

**Fig. 5:**
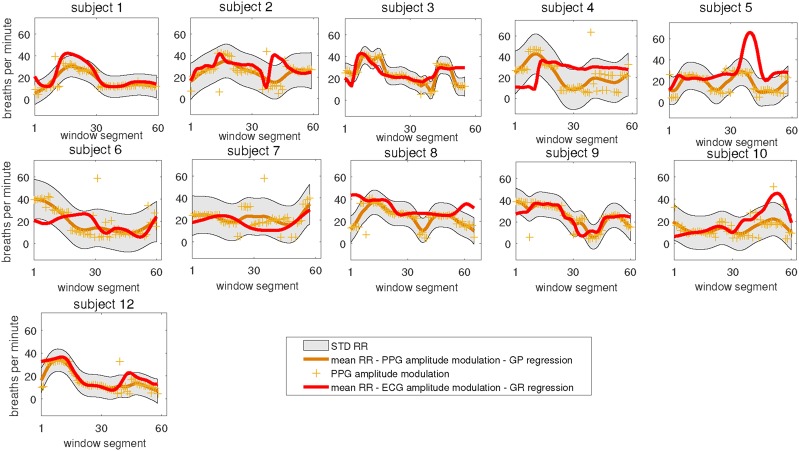
Estimated RR from PPG and ECG signals using amplitude modulated signals. Then mean RR estimates from ECG and PPG signals are shown in bold after applying GP regression

**Fig. 6:**
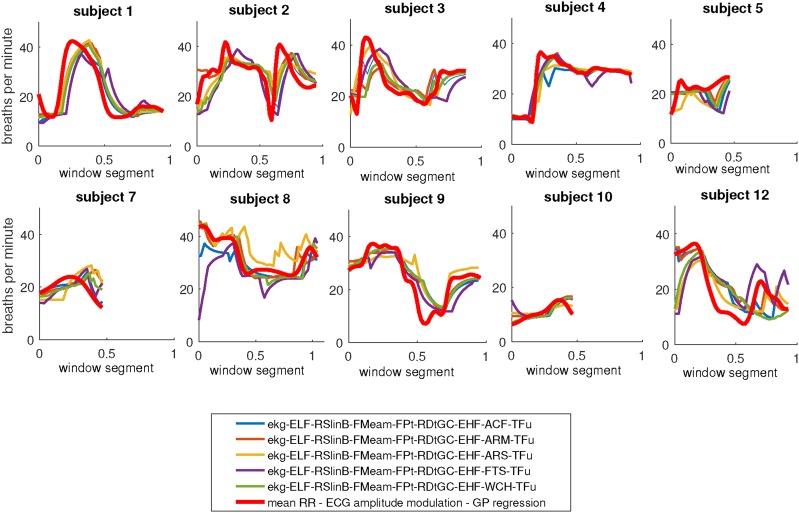
Estimated RR using ECG signals extracting amplitude modulated signals from RR toolbox [21] and AR spectrum based method [23]. The techniques used in RR toolbox are briefly noted as ELF: eliminates very low frequencies from resampled respiratory, RSlinB: resampling respiratory signal using linear interpolation, FMe: measures features from peak and through values, am: amplitude modulation, FPt: Fiducial point identification, RDt: R-spike detection, GC: ECG Beat detector, EHF: eliminates very high frequencies from signals, ACF: Autocorrelation Function, ARM: AR spectral analysis using the median spectrum for orders 2–20, ARS: AR spectral analysis, FTS: Fourier Transform, WCH: Welch Periodogram, and Tfu: fuses RR estimates using temporal fusion [21]

**Fig. 7:**
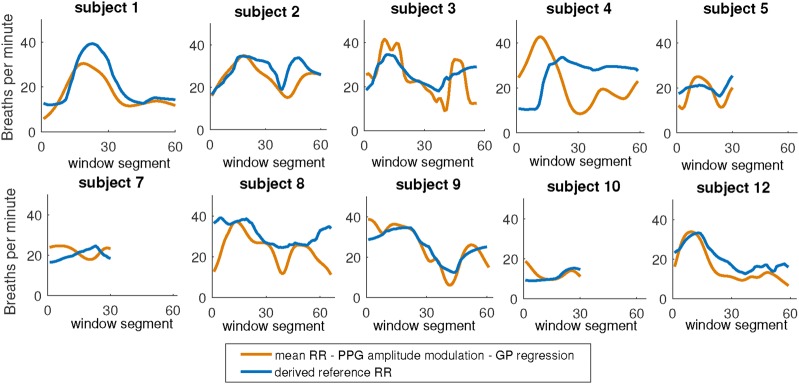
RR estimates from the proposed PPG based method compared to derived reference RR

The results (mean and standard deviation of RR estimates from PPG and mean RR estimates from ECG) are shown in Fig. 5, demonstrating a good agreement between ECG and PPG-based RR estimates. For each plot, consecutive window segments correspond to 4 s window step sizes where each window is of 64 s long. Similar settings have been used for Figs. 6 and 7. In this figure, overall trends of RR variations are preserved for most subjects motivating detailed analysis using other methods.

### 3.3 Deriving reference data

Since they are known to produce more accurate RR estimates, ECG signals have been used to derive the reference data. Using only amplitude modulations, the RR toolbox [21] has been applied to the ECG signals. The toolbox created the RR estimates using 12 methods. Of these 12, five methods which produced consistent results with low variance in output RR estimates were selected as detailed in Fig. 6. In addition, after manual peak detection of the ECG signals, the AR spectrum based method [23] has been applied using amplitude modulations, with GP applied in the final stage (see Fig. 6). For many subjects, the outputs of those five methods from the RR toolbox are in a strong agreement with RR estimates from the AR spectrum based method in [23], as shown in Fig. 6. We carefully manually labelled the ECG signals and applied AR spectrum based method to estimate RR. Therefore, as noted above, the RRs are calculated separately from manually annotated ECG signals followed by applying AR spectral analysis on resulted amplitude modulated signal and also from processed PPG signals after motion reduction using our proposed technique. As shown in Fig. 5, similar trends of variations of RR for most subjects especially subjects 1, 3, 9 and 12 are obtained. Additional RR estimates produced by the RR toolbox with consistent results are used to strengthen the reference RR. The basis for selection of the RR toolbox methods, that have been already validated comprehensively for RR estimation during rest, is observing the similar trend of RR variations with our PPG and ECG based analysis. The subjects were running on the treadmill with varying speed in which variations in HR for almost all subjects were observed. We removed those methods from RR toolbox which produced constant RR throughout the whole experiment or values less than < 10 bpm for several consecutive windows.

Considering the results shown in Fig. 6, a number of subjects are selected which show good agreement between RR estimates from ECG signals. The selected subjects are (1, 2, 3, 4, 5, 7, 8, 9, 10, and 12). For subjects 5, 7 and 10, only the first half of the RR estimates are in close agreement. For the selected subjects and segments, the average of all RR estimates from 5 of the RR toolbox methods and the AR spectrum based method is used as the reference data (see Fig. 7). The reference data is then used to compare and validate the PPG-based results.

### 3.4 Comparison of PPG and ECG based RR estimates

The RR estimates obtained using the proposed techniques which applied the AR spectrum based method onto the reconstructed PPG signals are compared to the ECG-based results from the RR toolbox [21] (see Fig. 7). Considering this figure, there is a good agreement for subjects 1, 2, 3, 5, 9 and 12, where the similar variations in RR estimates can be clearly observed. The worst results relate to subject 4. For the selected data segments, using an average of all ECG-based methods from 11 subjects (Figs. 6 and 7), the proposed PPG based method produces the worst estimates for only one subject (number 4) while for the other subjects the results demonstrate an acceptable level of agreement between RR estimates.

The RR toolbox [21] has also been applied to the PPG signals. Eight methods from the toolbox, including four whose results are similar to the reference data for most segments, are selected for comparison. The mean absolute error (MAE) of breaths per minute is calculated as

(8) $${\rm MAE} = \displaystyle{1 \over c}\sum\limits_{i = 1}^c \vert b\lpar i\rpar - \hat b\lpar i\rpar \vert \eqno\lpar 8\rpar $$

where *c* is the number of RR estimates for each subject (i.e. the number of segments), $b\lpar i\rpar $ is estimated breaths per minute and $\hat b\lpar i\rpar $ is derived breaths per minute using reference data for the *i*th segment. The standard error (SE) of mean is calculated as

(9) $${\rm SE} = \displaystyle{S \over {\sqrt c }}\eqno\lpar 9\rpar $$

where *S* is the standard deviation of the absolute difference between estimations and reference data ($\vert b - \hat b\vert $). The errors of RR estimates were calculated in terms of mean absolute errors and standard deviations of the errors between estimates from each PPG method and the reference data, for each subject. The PPG methods tested include the proposed method and eight selected methods from the RR toolbox. The MAE and 1.96 SE are calculated for each subject and shown in Table 1. The mean error over all subjects was calculated as 5.53, 6.32, 6.70, 6.63, 6.69, 31.40, 9.34, 10.2 and 9.70 breaths per minute for the proposed PPG-based technique, PPG^1^, PPG^2^, PPG^3^, PPG^4^, PPG^5^, PPG^6^, PPG^7^ and PPG^8^. The selected PPG based methods are

PPG^1^: ppg-ELF-RSlinB-FMeam-FPt-PDTIMS-EHF-CtA-TFu
PPG^2^: ppg-ELF-RSlinB-FMeam-FPt-PDTIMS-EHF-CtO-TFu
PPG^3^: ppg-ELF-RSlinB-FMeam-FPt-PDTIMS-EHF-PZX-TFu
PPG^4^: ppg-ELF-RSlinB-FMeam-FPt-PDTIMS-EHF-ZeX-TFu
PPG^5^: ppg-ELF-RSlinB-FMeam-FPt-PDTIMS-EHF-ARP-TFu
PPG^6^: ppg-ELF-RSlinB-FMeam-FPt-PDTIMS-EHF-ARS-TFu
PPG^7^: ppg-ELF-RSlinB-FMeam-FPt-PDTIMS-EHF-FTS-TFu
PPG^8^: ppg-ELF-RSlinB-FMeam-FPt-PDTIMS-EHF-WCH-TFu

All abbreviations are explained and detailed in [21] and briefly noted in the caption of Fig. 6. We observed that the method PPG^1^ has the lowest absolute error among the RR toolbox methods, but note that it does not follow the variation in RR estimates well. For many subjects, there is a bias even when the average errors are still low. For example, for subject 9, PPG^1^ method produced almost steady RR around 20 bpm leading still relatively a low average error but it failed to produce variations observed in the reference RRs increasing into 40 bpm and then decreasing to about 15 bpm. In contrast, the proposed PPG method in Fig. 7 matches the variation in RR estimate of the reference data much better for many subjects, although its overall MAE is only 1 bpm less than the lowest absolute error produced by the RR toolbox.

**Table 1:** Error, in breaths per minute calculated as [MAE ± 1.96 × SE (standard error)] for selected subjects

Method (mean)	Subject 1	Subject 2	Subject 3	Subject 4	Subject 5	Subject 7	Subject 8	Subject 9	Subject 10	Subject 12
proposed PPG (5.53)	$4.36 \pm 0.87$	$4.23 \pm 1.09$	$5.34 \pm 1.21$	$15.03 \pm 1.79$	$4.13 \pm 0.76$	$4.53 \pm 0.80$	$7.46 \pm 1.69$	$3.02 \pm 0.70$	$2.33 \pm 0.94$	$4.79 \pm 0.65$
PPG^1^ (6.32)	$6.02 \pm 1.64$	$6.96 \pm 0.99$	$5.50 \pm 0.80$	$5.53 \pm 0.39$	$2.35 \pm 0.60$	$3.73 \pm 0.94$	$14.84 \pm 0.93$	$6.51 \pm 1.02$	$8.26 \pm 0.75$	$2.50 \pm 0.47$
PPG^2^ (6.70)	$6.12 \pm 0.82$	$3.94 \pm 0.87$	$2.87 \pm 0.57$	$5.57 \pm 1.92$	$9.13 \pm 0.83$	$7.66 \pm 1.08$	$5.61 \pm 0.62$	$5.84 \pm 0.81$	$15.61 \pm 1.15$	$4.66 \pm 0.60$
PPG^3^ (6.63)	$6.98 \pm 1.50$	$8.08 \pm 0.99$	$6.22 \pm 1.01$	$8.47 \pm 0.47$	$2.69 \pm 0.66$	$4.43 \pm 1.12$	$12.65 \pm 0.80$	$5.18 \pm 1.12$	$9.48 \pm 0.76$	$2.06 \pm 0.39$
PPG^4^ (6.69)	$6.95 \pm 1.56$	$8.33 \pm 1.01$	$6.30 \pm 0.99$	$8.49 \pm 0.48$	$2.69 \pm 0.64$	$4.36 \pm 1.06$	$12.71 \pm 0.79$	$5.27 \pm 1.11$	$9.76 \pm 0.89$	$1.99 \pm 0.38$
PPG^5^ (31.40)	$33.42 \pm 2.36$	$25.60 \pm 1.53$	$30.88 \pm 1.43$	$30.84 \pm 1.90$	$34.07 \pm 2.43$	$28.80 \pm 5.16$	$20.38 \pm 2.58$	$28.89 \pm 1.80$	$45.44 \pm 1.03$	$35.68 \pm 1.61$
PPG^6^ (9.34)	$9.46 \pm 2.44$	$12.81 \pm 1.98$	$12.57 \pm 1.82$	$16.02 \pm 1.92$	$7.66 \pm 1.20$	$6.48 \pm 1.56$	$9.99 \pm 1.18$	$12.05 \pm 2.07$	$1.34 \pm 0.52$	$5.02 \pm 0.65$
PPG^7^ (10.2)	$10.48 \pm 2.68$	$14.67 \pm 1.84$	$14.03 \pm 1.84$	$18.14 \pm 1.95$	$6.17 \pm 1.56$	$5.54 \pm 1.41$	$11.97 \pm 1.63$	$13.18 \pm 2.47$	$1.52 \pm 0.41$	$4.49 \pm 0.86$
PPG^8^ (9.70)	$9.14 \pm 2.42$	$13.88 \pm 1.99$	$13.66 \pm 1.86$	$16.88 \pm 1.91$	$5.72 \pm 1.55$	$6.29 \pm 1.70$	$12.18 \pm 1.77$	$13.12 \pm 2.24$	$1.38 \pm 0.33$	$4.77 \pm 0.75$

The average runtime for each subject has been obtained as 2.8 s on an Intel@ Xeon@ Processor E5-1630 v3 (10M Cache, 3.70 GHz). The implemented code can be further optimised to be applicable in wearable devices.

## 4 Discussion

In this Letter, a new approach is proposed to estimate RR from motion contaminated PPG signals. Estimation of the RR during motion has been previously limited to ECG based analysis. The proposed method has shown to be very effective in motion reduction of PPG signals enabling estimation of RR and instantaneous HR.

Based on the proposed method, first, simultaneous accelerometer signals are used as inputs to adaptive filters. These are applied to raw PPG signals to reduce motion artefact and produce a clear spectrum containing the dominant HR frequency. Then the HT is used to reconstruct PPG signals in the time domain, using extracted phase modulations, peaks and onsets from this reconstructed signal are tracked back to the raw signal and used to derive the respiratory-induced amplitude modulation. RR is estimated from this modulation using an AR spectrum based method. GP regression has been applied to smooth the RR estimates.

Aside from RR estimation, the proposed technique has other potential uses, e.g. peaks in reconstructed PPG signals can be used to derive instantaneous HR from PPG, which can be used to study HRV. Here a simple window has been used to locate back peaks in the raw PPG signal, in future studies, this could be made more reliable by tracking the highest energy frequency in the time-frequency domain.

Some advantages of our method are that there are fewer parameters to set and the PPG signal reconstruction step provides new insight into the analysis of HRV. It also lends itself well to extracting respiratory modulations for use in the AR method. Extraction of the respiratory signal from PPG signals during motion is a challenging task. The proposed method in this Letter aimed to combine various techniques which would reduce motion artefact and better estimate RR. This can provide a basis for future studies on estimation of RR and HRV from motion contaminated PPG signals.

The limitation of this Letter is the lack of ground truth data. However, six different ECG-based methods which produced similar results are selected, with an average of these RR estimates used as the ground truth estimates. Although the PPG-based method proposed in this Letter produced more accurate estimates of the RR than those from the RR toolbox (when compared to ECG-derived ground truth data), the minimum difference in estimates is about 1 bpm. This also shows very good agreement between our proposed PPG based method and the nine selected methods (five using ECG-based methods and four using PPG-based methods). The variation of RR estimates over time has been preserved by the proposed PPG-based technique, while the final average difference between our method and the best method from the RR toolbox differs only by about 1 bpm.

This Letter provides a new platform for processing of PPG signals recorded during motion. In future studies, large datasets of signals from wearable sensors, e.g. at home environments could be recorded where the information of simultaneous accelerometer data can assist to improve the estimation of various physiological parameters. RR estimation during intense physical exercise has important applications in sports monitoring to provide athletes with crucial information to manage their training levels. On the other hand, monitoring of RR for patients during walking or running can help them to control their activity levels. Since the dataset analysed in this Letter is publicly available, we challenge future studies to process the PPG signals exploiting new RR based methods and compare the estimation of RR during motion. Another related dataset has been provided in [34] which includes gyroscope information which can allow separation of acceleration due to gravity. Reliable ground truth data is indeed necessary for detailed validation of estimated RR which needs to be considered in future studies.

## 5 Funding and declaration of interests

This work was supported by the U.K. Engineering and Physical Sciences Research Council under Grant EP/N024966/1. It was also supported by the Wellcome/EPSRC Centre for Medical Engineering at Kings College London [WT 203148/Z/16/Z].
